# Implicit expectation modulates multisensory perception

**DOI:** 10.3758/s13414-022-02460-z

**Published:** 2022-03-01

**Authors:** Mick Zeljko, Philip M. Grove, Ada Kritikos

**Affiliations:** School of Psychology, The University of Queensland, St. Lucia, QLD 4072 Australia

**Keywords:** Expectation, Perceptual ambiguity, Multisensory integration

## Abstract

Stimulus statistics can induce expectations that in turn can influence multisensory perception. In three experiments, we manipulate perceptual history by biasing stimulus statistics and examined the effect of implicit expectations on the perceptual resolution of a bistable visual stimulus that is modulated by sound. First, we found a general effect of expectation such that responses were biased in line with the biased statistics and interpret this as a bias towards an implicitly expected outcome. Second, expectation did not influence the perception of all types of stimuli. In both Experiment 1 and Experiment 2, integrated audio-visual stimuli were affected by expectation but visual-only and unintegrated audio-visual stimuli were not. In Experiment 3 we examined the sensory versus interpretational effects of expectation and found that contrary to our predictions, an expectation of audio-visually integrated stimuli was associated with impaired multisensory integration compared to visual-only or unintegrated audio-visual stimuli. Our findings suggest that perceptual experience implicitly creates expectations that influence multisensory perception, which appear to be about perceptual outcomes rather than sensory stimuli. Finally, in the case of resolving perceptual ambiguity, the expectation effect is an effect on cognitive rather than sensory processes.

## Introduction

The mechanisms underlying perceptual disambiguation are a central topic in sensory neuroscience (Parise & Ernst, 2017), and a common view is that, rather than being passively stimulus driven, perception is an active inferential process (Wang et al., 2013). Sensory input provides the brain with information that reflects the current state of the world, and prior knowledge, gained through experience, provides the brain with information about how the world works (e.g., Gekas et al., 2015; Gilbert & Sigman, 2007; Kersten et al., 2004; Kornmeier et al., 2009; Maloney et al., 2005; Summerfield & Egner, 2009; Wang et al., 2013). While the sensory input reflects the state of the world, it always underspecifies it; sensory information is varyingly noisy, incomplete, and weak, and so in general, it is ambiguous (Parise & Ernst, 2018; Urgen & Boyaci, 2021; Zeljko et al., 2019). However, perceptual decisions are reached without the impression of ambiguity as the incoming sensory information is interpreted within a framework of prior knowledge (e.g., Ernst & Bülthoff, 2004; Gilbert & Sigman, 2007).

Prior knowledge can manifest in various forms. Most fundamentally, context, or the broader perceptual environment, can bias perceptual decisions in favour of interpretations of sensory input that are known to be contextually consistent (Biederman et al., 1982; Bruner & Minturn, 1955). For example, Biederman et al. (1982) found that object detection in a scene was impaired when the object was unlikely in that particular scene, located in an inappropriate position in the scene, or was too large or too small relative to the other objects in the scene. Additionally, prior knowledge in the form of learned associations have been shown to bias perceptual decisions regarding ambiguous stimuli (Einhäuser et al., 2017), priming (e.g., Bugelski & Alampay, 1961; Intaitė et al., 2013; Ouhnana & Kingdom, 2016), and serial dependence (e.g., Brascamp et al., 2010; Pearson & Brascamp, 2008; Zeljko & Grove, 2021).

More generally, perceptual experience can create expectations about what is likely in the current sensory environment (Summerfield & Egner, 2009). In a visual search task, Gekas et al. (2015) had participants identify the presence and location of low contrast dots positioned at any of 12 points around the circumference of a circle. Expectations were manipulated by dividing them into two groups of six locations each: a frequent group and a nonfrequent group. The dot would appear with 70% probability at a location in the frequent group and 30% probability at a location in the nonfrequent group. They found that the stimulus distribution was quickly learned (in around 5–8 min of stimulus presentation), resulting in improved performance, although this was accompanied by more false alarms at the high probability locations. Relevant to the present study, expectations have been shown to affect multisensory integration (for a review, see Shams & Beierholm, 2010) and biasing stimulus statistics modulates expectations regarding multisensory stimuli (e.g., Gekas et al., 2015; Van Wanrooij et al., 2010).

Van Wanrooij et al. (2010) examined orienting responses to audio-visual stimuli in which the probability of crossmodal spatial alignment varied between experiments. The authors found that stimulus statistics altered head saccades such that reaction times were faster in blocks consisting of only spatial aligned stimuli compared with blocks that included misaligned stimuli. They interpreted this as stimulus history dynamically updating expectations of alignment which in turn affected the strength of multisensory integration. Gau and Noppeney (2016) demonstrated that congruency expectations built up over time and significantly modulated the integration of visual and auditory McGurk stimuli. Participants viewed McGurk stimuli that were embedded in blocks of either phonologically congruent or incongruent audio-visual syllables and were more likely to experience the illusory McGurk percept in congruent than incongruent blocks. That is, an expectation of incongruency blocked the multisensory integration required for the illusion and this was accompanied by differential activity in the left inferior frontal sulcus.

Recent work has considered the influence of prior knowledge in the perception of ambiguous multisensory stimuli using the stream-bounce display, an ambiguous motion sequence in which two identical targets moving along intersecting trajectories are typically seen to either stream past or bounce off one another. Typically, these stimuli are bistable, and observers report a mix of stream and bounce percepts. The main finding is that stimulus manipulations at the point of target coincidence modulate this bistabilty (Sekuler et al., 1997; Zeljko & Grove, 2017a, 2017b). For example, the presence or absence of a brief sound at the point of coincidence of the targets biases responses such that sounds are reliably associated with increased bounce reports (Sekuler et al., 1997). A more recent finding is that other factors, occurring well before the critical point of target coincidence also modulate responses.

Results from our program of research so far indicate that non-sensory factors, such as perceptual history and expectation play a significant role in the resolution of these displays. For example, Grove et al. (2016) examined the influence of pre-coincidence factors using a stream-bounce display in which the targets moved along horizontal, intersecting trajectories as usual, but in this case the trajectories were slightly vertically offset. The offset targets always objectively streamed, so to report a bounce, an observer would need to accept that the targets had undergone a small vertical shift at the point of coincidence. That is, at the point of coincidence, the target on the upper trajectory must have instantaneously shifted to the lower trajectory and vice versa. The researchers examined the effect of introducing similar trajectory shifts prior to the point of coincidence. They found that pre-coincidence shifts were associated with increased reported bouncing, and higher number of pre-coincidence shifts associated with greater reported bouncing. Grove et al. concluded that trajectory shifts prior to coincidence were creating expectations that primed perceptual inference to modulate subsequent perceptual decisions by making shifts at coincidence, in effect, an acceptable perceptual interpretation.

Next, Zeljko et al. (2019) implemented a modified version of the stream-bounce display that tracked responses to stream-bounce stimuli dynamically over the entire course of the motion sequence rather than collecting a subjective report after the fact. Participants used a trackpad to control a cursor to track a stream-bounce target actively from the beginning to the end of its trajectory. Tracking speed was recorded throughout as the dependent variable. In addition to the usual finding that a sound at coincidence was associated with increased bounce responses, there was also a significant difference in pre-coincidence tracking speed for bounce compared with stream responses. Specifically, tracking speeds were significantly slower starting 500 ms before the critical point of coincidence for bounce compared with stream responses. So, bounce responses were associated with a combination of a sound at coincidence and a slowing of tracking speed before coincidence. The authors suggested that the behavioural response reflected a cognitive expectation of a perceptual outcome that then biased both action and the interpretation of sensory input to favour that forthcoming percept.

Finally, Zeljko and Grove (2021) examined perceptual disambiguation and crossmodal interactions by considering the effect of recent perceptual history on stream-bounce perception. The authors compared groups of naïve stream-bounce observers first exposed to either only audio-visual or only visual-only stream-bounce stimuli, and then to mixed audio-visual and visual-only stimuli. After exposure to audio-visual stimuli, visual-only stimuli were associated with reduced bounce responses, while after exposure to visual-only stimuli, audio-visual stimuli were associated with increased bounce responses. Further, there was a serial dependence in responses in which both audio-visual and visual-only stimuli were processed with a bias to the interpretation of the previous stimulus regardless of its modality. The authors took these findings as support for top-down interpretational influences in stream-bounce perception that rely heavily on recent perceptual history.

In addition to our recent findings, other work using audio-visual stream-bounce stimuli has found that information provided before target overlap can influence outcomes. For example, Grassi and Casco (2010) found that a semantically bounce like sound resulted in a greater proportion of bounce responses than semantically non-bounce like sounds, but only when the sounds were presented 200 ms before the targets coincided.

The reviewed stream-bounce work suggests that factors occurring well before the critical point of target coincidence can modulate perceptual outcomes in stream-bounce perception, and stimulus statistics can modulate expectations. Here, we apply a biased statistics paradigm to stream-bounce stimuli to ascertain if directly manipulating the audio-visual statistics of stream-bounce events influences the likelihood of multisensory integration and therefore the likelihood of bounce responses to audio-visual stimuli. Our aim is to test if perceptual experience, in the form of biased stimulus statistics, creates an implicit expectation that modulates the resolution of ambiguity in visual-only and audio-visual perception. We conduct our investigation using a stream-bounce stimulus but employ a novel biasing manipulation to induce implicit expectations. In a standard stream-bounce design, participants are typically presented with a mix of visual-only and audio-visual motion sequences and tasked with making a subjective report as to whether the targets appeared to stream or bounce. In our first experiment, we employ this approach but additionally vary the shade of the targets such that on half the trials they are both white and on half they are both black. While overall we split visual-only and audio-visual trials 50/50, our expectation manipulation rests on the proportion of audio-visual to visual-only trials for each shade: for one shade, 80% of trials are audio-visual and 20% visual-only, while for the other shade, the reverse. Our hypothesis regarding this manipulation is that one shade will therefore be more strongly (but implicitly) associated with sounds, or bouncing, or both, and the other shade less so. We refer to the shade with 80% audio-visual trials as “high bounce expectation targets” and the shade with 80% visual-only trials as “low bounce expectation targets”.

We hypothesise that, if implicit expectations modulate perceptual outcomes, then we expect a positive effect such that there will be a greater proportion of bounce (vs. stream) responses for high bounce expectation targets compared with low bounce expectation targets. To preface our results, we found an overall effect of expectation, but also an interaction whereby expectation positively influenced the perceptual resolution of audio-visual stimuli but not visual-only stimuli. Our subsequent experiments were developed specifically to follow up this finding by considering audio-visual stimuli only. To vary the likelihood of bounce responses with only audio-visual stimuli, we vary the temporal offset between the presentation of the sound and the visual coincidence of the targets. This approach is based on the reliable stream-bounce finding that sounds with small offsets from visual coincidence elicit more bounce responses than those with large offsets (Sekuler et al., 1997; Watanabe & Shimojo, 2005). To manipulate expectation, we again present either white or black targets, but instead of varying the proportions of audio-visual versus visual-only for each shade, we vary the proportion of trials with small versus large audio-visual offsets.

## Method

### Participants

One hundred and sixty undergraduate students (50 male, 22.0 ± 6.5 years) from the University of Queensland participated in three separate experiments in return for course credit. All participants reported normal hearing and normal or corrected to normal vision and all were naïve as to the purpose of the experiment. All experiments were cleared in accordance with the ethical review processes of the University of Queensland and within the guidelines of the National Statement on Ethical Conduct in Human Research.

While stream-bounce effects are typically large (d > 1), effects of implicit expectation are likely smaller, so, for Experiment 1, we assumed a medium effect size. We conducted a sensitivity analysis using G*Power and found that two tailed dependent means t-tests with 30 participants and an alpha of 0.05 could detect an effect size of 0.53 with 80% power (Faul et al., 2007). Given that our expectation manipulation in Experiment 2 is much more subtle than in Experiment 1 (i.e., audio-visual offsets rather than sound or no-sound), we anticipated that any effect of expectation might also be smaller. For Experiment 2 we assumed a small effect size and found that 80 participants and an alpha of 0.05 could detect an effect size of 0.32 with 80% power. Finally, while Experiment 3 used the same subtle expectation manipulation as Experiment 2, we anticipated a larger effect on the sensory measures used in Experiment 3 since they are calculated based on responses in all of the duration conditions and not just a subset of them (as in Experiment 2). Assuming an effect size between those for Experiments 1 and 2 we found that 50 participants and an alpha of 0.05 could detect an effect size of 0.41 with 80% power.

### Apparatus and stimuli

Stimuli were generated on a Mac mini (2.5 GHz Intel Core i5 processor with 4 GB 1600 MHz DDR3 memory, an Intel HD Graphics 4000 1024 MB graphics chip and running OS X 10.9.5) using MATLAB (R2015b, 2015) and the Psychophysics Toolbox extensions (V3.0.11) (Brainard, 1997; Kleiner et al., 2007). Visual stimuli were viewed on an Apple Thunderbolt Display (resolution 2,560 × 1,440) and sounds were presented via a set of Sony MDR-XB450 headphones.

Visual stimuli consisted of two identical targets (either two black discs OR two white discs on a grey background) separated horizontally about the midline of the display and viewed from approximately 80 cm. Each target was 0.8° in diameter and there was a small black fixation cross in the centre of the display. The target centres were positioned 0.8° above the horizontal midline of the display.

Motion sequences consisted of 121 frames presented at a frame rate of 60 Hz, and consisted of a central coincidence frame, at which the targets completely overlapped, and 60 frames before and 60 frames after coincidence, providing a sequence lasting 2 s. Only the fixation cross was visible before trial initiation, after which the targets appeared (frame 1) then immediately approached the screen midline on a horizontal trajectory (frames 1–60), coincided (frame 61), continued their motion (frames 62–120) to the end point of the sequence (frame 121), and then disappeared. The targets were horizontally displaced by 0.08° with each frame change resulting in a constant apparent speed of 5°/s. The fixation cross remained visible throughout the entire sequence.

The auditory stimulus was a 15 ms 800 Hz sine wave tone, offset modulated with an exponential decay and sampled at 44.1 kHz. Sound onset was not modulated. The average sound pressure level of the tone was approximately 65dB SPL measured at the headphone earpiece (measured with a Lutron SL-4012 sound level meter). Ambient sound level was approximately 45dB SPL. If and when the auditory stimulus was played depended on the experiment and the specific details are provided below.

### Procedure

Participants were seated unrestrained, approximately 80 cm in front of the display and the initiated the first trial by pressing the “space” bar on a computer keyboard. After observing the entire motion sequence, they used the left and right arrows on a computer keyboard to respond (the response options depended on the experiment and are described in detail below). Responses were untimed and the next trial was automatically initiated following the response.

### Design

Each of the three experiments was a fully within-subjects design with two expectation conditions (high bounce or low bounce) and a number of sound conditions (depending on the experiment). In Experiment 1 the auditory stimulus was either present (and played when the targets coincided) or absent to give two sound conditions (no-sound or sound). In Experiments 2 and 3 the auditory stimulus was always present but was varyingly offset from target coincidence to give eight sound offset conditions (-300 ms, -183 ms, -100 ms, -50 ms, 50 ms, 100 ms, 183 ms, 300 ms). All sound and expectation conditions were randomly varied within each experiment.

Expectation was manipulated by stimulus statistics. The pairing of different sound conditions with specific target shades (black or white) was biased such that 80% of the sound conditions that are typically associated with more bouncing were paired with one target shade (high bounce expectation targets) and 80% of the sound conditions that are typically associated with less bouncing were paired with the other target shade (low bounce expectation targets). In each experiment the specific target shade for the high bounce and low bounce conditions was counter-balanced across participants. Critically, participants were not informed of the sound/shade biasing, making the expectation manipulation implicit.

## Experiment 1: Perceptual effects of Expectation 1

Experiment 1 was a 2 (sound: no-sound or sound) × 2 (expectation: high bounce or low bounce) within-subjects design to examine the perceptual effects of implicit expectation. There were 240 trials completed in a single block. In 50% of the trials, both targets were black and in the other 50% both targets were white. In 50% of the trials, the sound was present (sound) and in 50% it was absent (no-sound). To manipulate expectation, the audio-visual stimuli were paired such that for one target shade, the sound was present in 80% of the trials and absent in 20%, while for the other shade, the sound was present in only 20% of the trials and absent in 80%. The target shade associated with 80% sound was labelled the “high bounce” condition, and the target shade associated with 20% sound was labelled the “low bounce” condition based on the reliable stream-bounce finding that sound trials elicit more bounce responses than no-sound trials (Sekuler et al., 1997) (see Table 1 for the stimulus statistics matrix).

**Table 1:** Number of sound and no-sound trials for each Expectation condition

	Sound	No-sound	Total
High Bounce	96	24	120
Low Bounce	24	96	120
Total	120	120	240

## Results

**Fig. 1:**
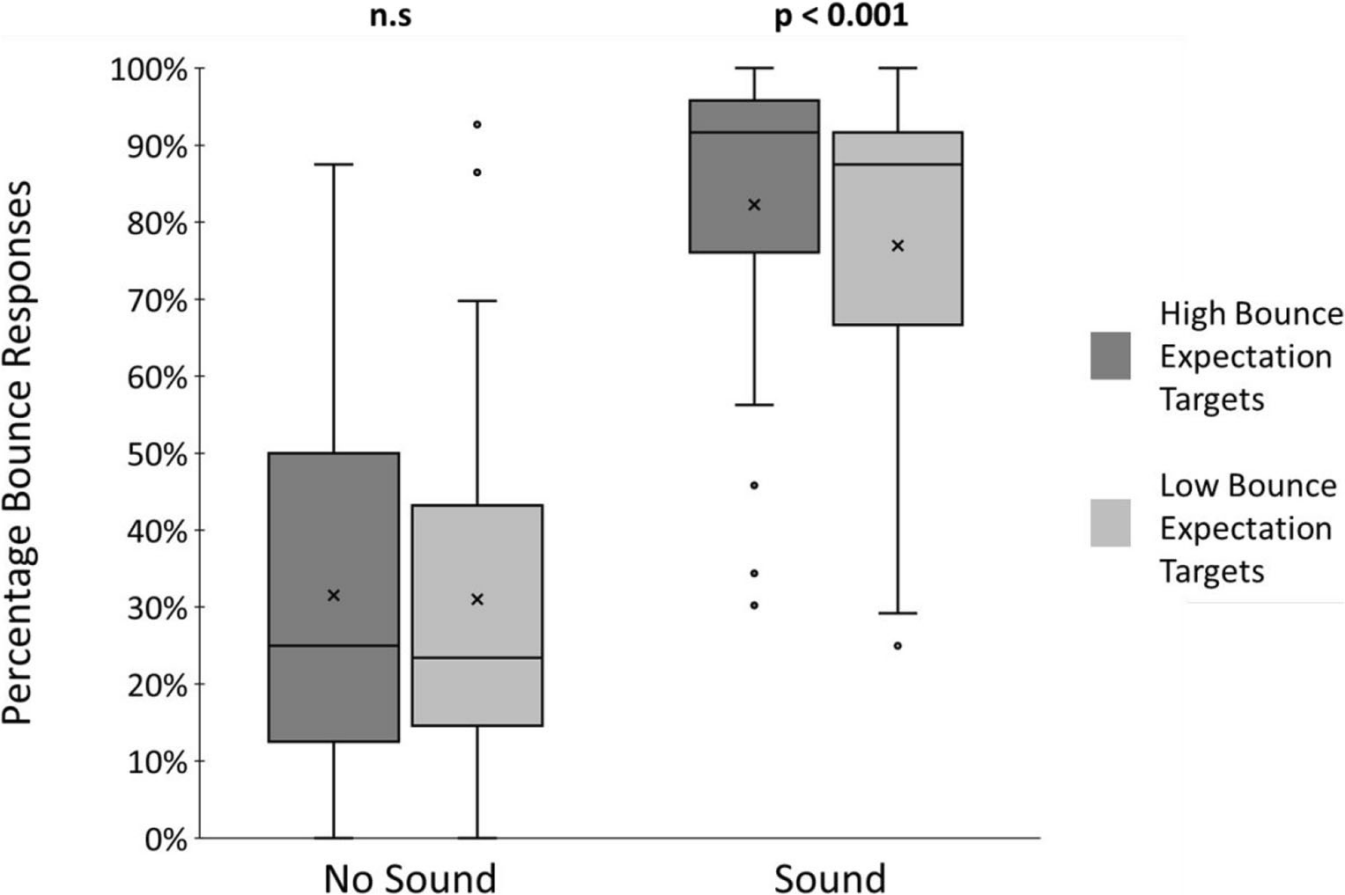
Group percentage of bounce responses (‘x’ indicates the group mean) for the high bounce (dark grey) and low bounce (light grey) expectation conditions in each of the sound conditions (no sound and sound)

We computed the percentage of trials yielding bounce responses for each participant in each of the four conditions (sound: no-sound or sound; expectation: high bounce or low bounce) and conducted a 2 (sound) × 2 (expectation) repeated-measures ANOVA on these data. Figure 1 shows the group mean responses and provides a reference for our comparisons and analyses.

We found a significant main effect of sound (*F*(1,29) = 113.33, *p* < 0.001, *η*_*p*_^*2*^ = 0.796), reflecting the usual stream-bounce effect, such that there was a significantly greater percentage of bounce responses to sound trials (*M* = 81%, *SE* = 4%) than to no-sound trials (*M* = 32%, *SE* = 4%). In terms of expectation, we first report a significant main effect of expectation (*F*(1,29) = 5.13, *p* = 0.031, *η*_*p*_^*2*^ = 0.150) whereby the percentage of bounce responses was significantly higher for high bounce expectation targets (*M* = 57%, *SE* = 3%) compared to low bounce expectation targets (*M* = 54%, *SE* = 4%). We followed up this finding by examining individual participant expectation effects to check for outliers and note that all individual effects were within three standard deviations of the mean. Second, we also found a significant interaction between expectation and sound (*F*(1,29) = 4.29, *p* = 0.047, *η*_*p*_^*2*^ = 0.129). Follow-up paired t-tests revealed that the effect of expectation was present only for sound trials (*M(High Bounce)* = 82%, *SE(High Bounce)* = 4%, *M(Low Bounce)* = 77%, *SE(Low Bounce)* = 4%, *t*(29) = 4.13, *p* < 0.001, *d* = 0.13) but not for no-sound trials (*M(High Bounce)* = 31%, *SE(High Bounce)* = 5%, *M(Low Bounce)* = 32%, *SE(Low Bounce)* = 4%, *t*(29) = 0.25, *p* = 0.807).

These findings support the hypothesis that implicit expectations exert an influence on responses to audio-visual stream-bounce stimuli. The effect size is small, but we note that the effect was established implicitly and rapidly with only brief exposure to the biased stimulus statistics (240 trials completed in under 10 min). Anecdotally, participants remained mostly unaware of the biased stimulus statistics underlying the expectation manipulation. The fact that we observed no expectation related differences in bouncing for visual-only events suggests that the effect is not simply a case of associative learning. That is, if bounce responses were higher for the high bounce expectation compared with low bounce expectation targets independent of sound, it may be that one shade is simply associated more with bouncing than the other shade. The effect, however, seems to be related to the perceptual interpretation of multisensory stimuli.

Since the presence of a sound is strongly correlated with reported bouncing, it remains uncertain as to what precisely the expectation relates to. High bounce expectation targets might lead to an expectation of a forthcoming sound that increases the likelihood of a bounce response if there is a sound. Alternatively, high bounce expectation targets might lead to an expectation of a forthcoming bounce that increases the likelihood of a bounce response if there is a sound. This is an important distinction: the expectation may be sensory and relate to the stimulus, or it may be perceptual and relate to the ultimate interpretation of the stimulus. Experiment 2 addresses this uncertainty by fixing the stimuli to be all audio-visual stream-bounce stimuli. We vary the bounce likelihood by varying the timing of the sound relative to the coincidence of the visual targets.

## Experiment 2: Perceptual effects of Expectation 2 (temporal offsets)

Experiment 2 used only audio-visual stream-bounce stimuli but varied the offset of the sound relative to the coincidence of the visual targets. It was an 8 (audio-visual offset: -300 ms, -183 ms, -100 ms, -50 ms, 50 ms, 100 ms, 183 ms, 300 ms) × 2 (expectation: high bounce or low bounce) within-subjects design to examine sensory versus interpretational effects of expectation. There were 800 trials completed in four blocks with brief breaks in between blocks (the duration of the experiment was approximately 45 min). In 50% of the trials, both targets were black and in the other 50% both targets were white. There were 100 trials of each sound offset condition. To manipulate expectation, the audio-visual stimuli were combined such that for one target shade, the sound was present at a small offset magnitude (-100 ms, -50 ms, 50 ms, 100 ms) in 80% of the trials and at a large offset magnitude (-300 ms, -183 ms, 183 ms, 300 ms) in 20%, while for the other shade, the sound was present at a small offset magnitude in only 20% of the trials and a large offset magnitude in 80%. The target shade associated with 80% small magnitude offsets was labelled the “high bounce” condition, and the target shade associated with 20% small magnitude offsets was labelled the “low bounce” condition (see Table 2 for the stimulus statistics matrix).

**Table 2:** Number of trials for each sound offset (ms) in each expectation condition

	-300	-183	-100	-50	50	100	183	300	Total
High Bounce	20	20	80	80	80	80	20	20	400
Low Bounce	80	80	20	20	20	20	80	80	400
Total	100	100	100	100	100	100	100	100	800

## Results

Before considering the effect of implicit expectation on responses to stream-bounce stimuli, we first analysed our results to confirm that our expectation manipulation was working as anticipated and that overall, trials in which the sound had a small offset from visual coincidence resulted in a higher proportion of bounce responses than trials with large offsets. We computed the percentage of trials yielding bounce responses for each participant in each of two offset magnitude conditions (small offsets: ±50 ms and ±100 ms; large offsets: ±183 ms and ±300 ms), and these were the units for statistical analyses. A paired samples t-test confirmed that there was a greater percentage of bounce responses for small compared with large audio-visual offsets (*M(Small)* = 62%, *SE(Small)* = 2%, *M(Large)* = 48%, *SE(Large)* = 2%, *t*(79) = 8.69, *p* < 0.001, *d* = 0.29).

We next checked the expectation manipulation on an individual participant basis reasoning that, without an offset effect (the difference between average bounce responses for small versus large audio-visual offset), an implicit expectation could not occur. Seven participants showed a negative offset effect (that is, they reported a greater percentage of bounce responses for large offsets compared to small offsets) and so were removed from the dataset and excluded from further analyses. To be clear, we did not remove participants that failed to show the effect that we are investigating (an expectation effect), but removed those who failed to respond to the manipulation that would ultimately drive an expectation effect (i.e., the offset effect).

**Fig. 2:**
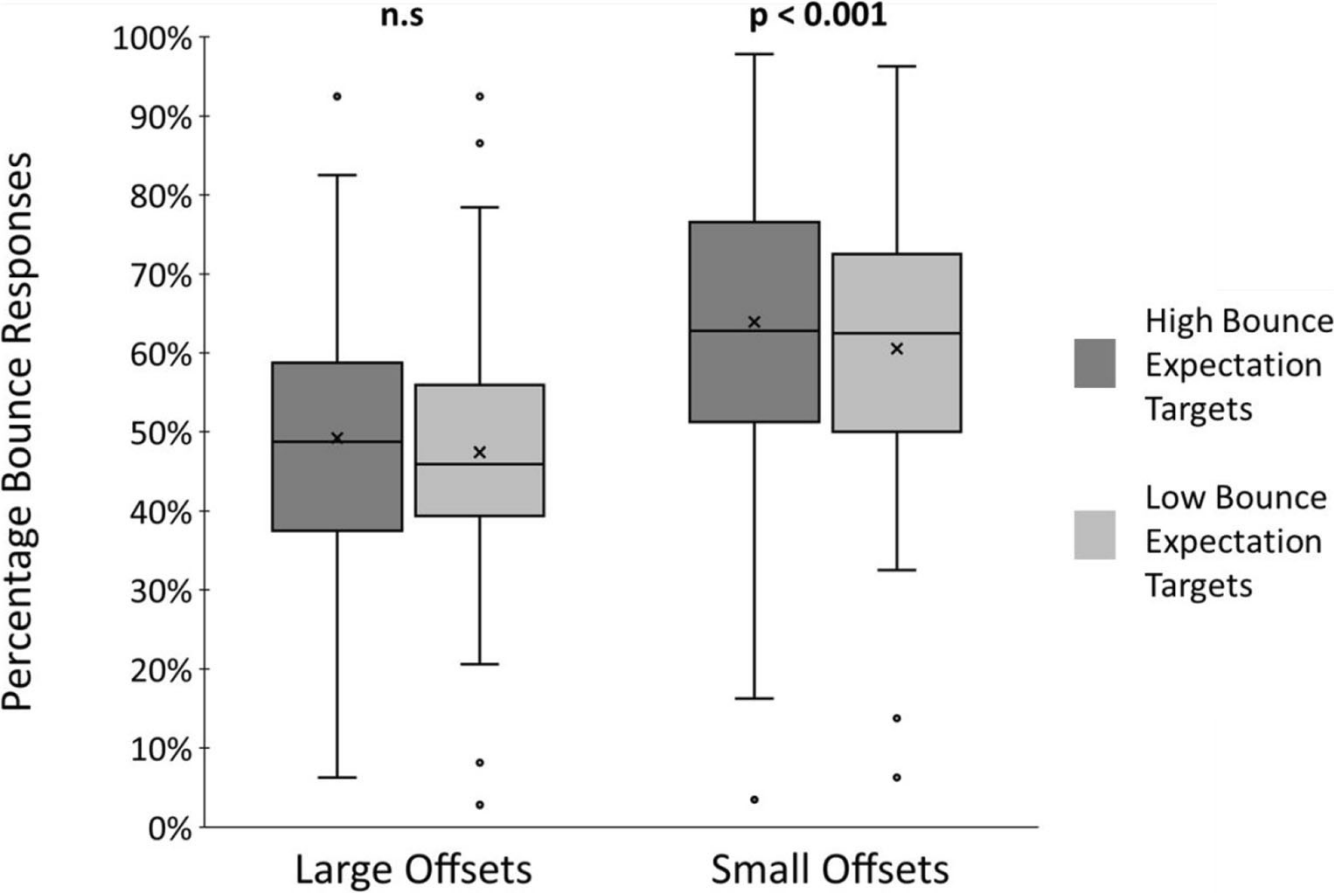
Group percentage of bounce responses (‘x’ indicates the group mean) for (**a**) high bounce expectation (dark grey) versus low bounce expectation targets (light grey) across all sound offsets, and for (**b**) high bounce expectation (dark grey) and low bounce expectation targets (light grey) for small and large sound offsets

Having demonstrated the offset effect in 73 out of 80 participants, we conclude that the expectation manipulation is valid so next consider the effect of implicit expectation. We computed the percentage of trials yielding bounce responses for each participant in each of the two expectation conditions (high bounce expectation and low bounce expectation) and each of two pooled offset conditions (small offsets: ±50 ms and ±100 ms; large offsets: ±183 ms and ±300 ms), and these were the units for statistical analyses. We first examined our data for outliers by considering overall expectation effects (i.e., collapsed across offset conditions) for individual participants and noted that two participants had expectation effects that were greater than three standard deviations from the mean (one substantially greater than the mean and one substantially less than the mean). These two participants were additionally removed from the dataset and excluded from further analyses leaving 71 participants in the final dataset. We conducted a 2 (expectation: high or low) × 2 (offset: small or large) repeated-measures ANOVA on the bounce response data. Figure 2 shows the group mean responses and provides a reference for our comparisons and analyses.

We found a significant main effect of offset (*F*(1,70) = 72.94, *p* < 0.001, *η*_*p*_^*2*^ = 0.510), reflecting the usual stream-bounce effect, such that there was a significantly greater percentage of bounce responses to small audio-visual offset trials (*M* = 62%, *SE* = 2%) than to large offset trials (*M* = 48%, *SE* = 2%). In terms of expectation, we first report a significant main effect of expectation (*F*(1,70) = 9.93, *p* = 0.002, *η*_*p*_^*2*^ = 0.124) whereby the percentage of bounce responses was significantly higher for high bounce expectation targets (*M* = 57%, *SE* = 2%) compared with low bounce expectation targets (*M* = 54%, *SE* = 2%). We followed up this finding by examining individual participant expectation effects to check for outliers and note that all individual effects were within three standard deviations of the mean. Second, we also found a significant interaction between expectation and sound (*F*(1,70) = 4.19, *p* = 0.044, *η*_*p*_^*2*^ = 0.056). Follow-up paired t-tests revealed that the effect of expectation was present only for small offset trials (*M(High Bounce)* = 64%, *SE(High Bounce)* = 2%, *M(Low Bounce)* = 61%, *SE(Low Bounce)* = 2%, *t*(70) = 3.68, *p* < 0.001, *d* = 0.07) but not for large offset trials (*M(High Bounce)* = 49%, *SE(High Bounce)* = 2%, *M(Low Bounce)* = 48%, *SE(Low Bounce)* = 2%, *t*(70) = 1.97, *p* = 0.053).

These findings further support the hypothesis that implicit expectations exert an influence on responses to audio-visual stream-bounce stimuli, and in fact replicate our Experiment 1 findings in that we see the same pattern of results in Figs. 1 and 2. Furthermore, the findings here clarify our Experiment 1 findings and suggest that the expectation appears to relate to perceptual rather than sensory factors. That is, given that all stimuli in Experiment 2 are audio-visual and the expectation effect was found only for trials with small audio-visual offsets, it appears that it is an implicit expectation of a bounce percept that is driving differential responses rather than an expectation of an audio-visual stimulus. These results additionally support our earlier contention that the expectation effect is more than a simple general biasing of response due to associating one target shade with a particular outcome.

The results of Experiments 1 and 2 support our hypothesis that implicit expectation exerts an influence on the perception of stream-bounce stimuli, and further suggest that it is an expectation of a perceptual outcome (namely, a bounce) that underlies this influence. The question remains, however, is expectation itself an effect on the integration of auditory and visual signals (i.e., is it sensory) or is it an effect on the perceptual interpretation of these signals (i.e., is it cognitive)? Experiment 3 addresses this by directly testing the effect of expectation on multisensory integration.

## Experiment 3: Sensory effects of expectation

Experiment 3 was identical to Experiment 2 except for the response requirement. The experiment was an 8 (offset: -300 ms, -183 ms, -100 ms, -50 ms, 50 ms, 100 ms, 183 ms, 300 ms) × 2 (expectation: high bounce or low bounce) within-subjects design, but rather than provide a subject report as to whether the targets streamed or bounced, participants are required to provide a temporal order judgement (TOJ) regarding whether the sound preceded or followed visual coincidence (the point where the two targets completely overlap). As in Experiment 2, responses were made with the left and right arrows following the presentation of the stimuli, but instead of responding stream (left) or bounce (right), participants responded whether the sound preceded (left) or followed (right) visual coincidence of the targets.

The aim of Experiment 3 was to examine sensory versus interpretational effects of expectation. Our hypothesis is that expectations of bounce events drive an increased likelihood of bounce responses by increasing the likelihood of audio-visual integration. Hence the TOJ task should be more difficult for high bounce expectation targets than for low bounce expectation targets. Accordingly, we expected a larger Just Noticeable Difference (JND) for high bounce expectation targets compared to low bounce expectation targets, but no effect on response bias as measured by the Point of Subjective Equality (PSE).

## Results

**Fig. 3:**
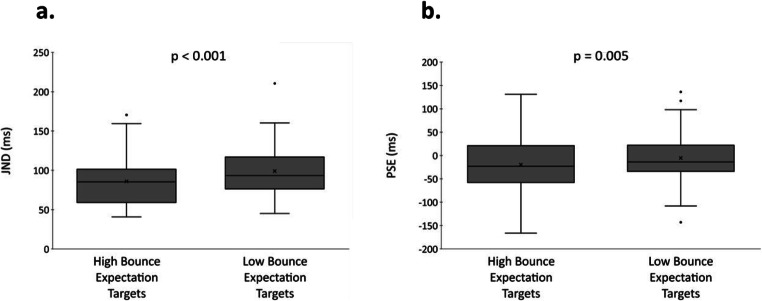
**a** Group JND (‘x’ indicates the group mean) for high bounce expectation versus low bounce expectation targets, and **b** group PSE (‘x’ indicates the group mean) for high bounce expectation versus low bounce expectation targets

We tabulated participant responses in terms of the sound occurring ‘before’ or ‘after’ visual coincidence for each of the eight audio-visual offsets separately for high and low bounce expectation targets. A cumulative normal function was then fit to each individual participant’s response data for each expectation condition to determine the mean and variance and these were used to determine individual JNDs and PSEs for each expectation condition. Participants whose JND was more than three standard deviations from the mean were identified as outliers and data from seven participants were subsequently removed from the dataset and excluded from further analysis. Figure 3 shows the group means and provides a reference for our comparisons and analyses.

Paired samples t-tests were conducted separately on the JND and PSE data and found significant effects for each that were contrary to our predictions. First, JND was lower for high bounce expectation targets compared with low bounce expectation targets (*M(High Bounce)* = 86.0, *SE(High Bounce)* = 5.0, *M(Low Bounce)* = 99.1, *SE(Low Bounce)* = 5.0, *t*(42) = -5.27, *p* < 0.001, *d* = 0.80). Second, there was a significant difference in bias such that the PSE for high bounce expectation targets was more auditory leading than for low bounce expectation targets (*M(High Bounce)* = -19.5, *SE(High Bounce)* = 11.7, *M(Low Bounce)* = -5.3, *SE(Low Bounce)* = 9.2, *t*(42) = -2.93, *p* = 0.005, *d* = 0.44).

## General discussion

Previous studies have shown that expectations can influence multisensory integration and that stimulus statistics can induce expectations. Using the stream-bounce display, our aim was to investigate if perceptual experience, in the form of biased stimulus statistics, creates an implicit expectation that can modulate the perceptual resolution of ambiguity.

Our first experiment considered visual-only and audio-visual stream-bounce stimuli and biased the presentation such that targets of one shade (say, black) were 80% audio-visual (high bounce expectation) while targets of another shade (white) were 80% visual-only (low bounce expectation). Findings were consistent with our hypothesis that implicit expectations modulate perceptual outcomes, and high bounce expectation targets were associated with an increased proportion of bounce responses. We further found an interaction such that expectation only affected multisensory but not unisensory stimuli. That is, audio-visual high bounce expectation targets had a greater proportion of bounce responses than audio-visual low bounce expectation targets. Conversely, visual-only high bounce expectation targets had statistically identical bounce responses as the visual-only low bounce expectation targets.

While there was a multisensory expectation effect, it was unclear whether it was an expectation of a forthcoming sound or an expectation of a forthcoming bounce that was driving the pattern of findings. That is, the expectation may be sensory and relate to the stimulus, or it may be perceptual and relate to the ultimate interpretation of the stimulus. We followed this up in Experiment 2, using only audio-visual stimuli and adjusting the likelihood of a bounce by varying the temporal offset between the presentation of the sound and the visual coincidence of the targets. We reasoned that an expectation of a percept (a bounce) would be associated with an analogous pattern of results for all audio-visual stimuli as for intermixed visual-only and audio-visual stimuli, but an expectation of a stimulus (a sound), would not. We again observed an expectation effect and high bounce expectation targets were associated with a greater proportion of bounce responses than low bounce expectation targets. Further, we observed an interaction analogous to that seen in Experiment 1. That is, the difference in bounce responses between high and low bounce expectation targets was only seen for high bounce likelihood targets (i.e., small temporal offsets, analogous to audio-visual stimuli in Experiment 1) and not for small bounce likelihood targets (i.e., large temporal offsets, analogous to visual-only stimuli in Experiment 1).

Finally, in Experiment 3, we examined if the expectation effect involves enhanced integration of auditory and visual signals (i.e., is it a sensory effect) or a modulated perceptual interpretation of these signals (i.e., is it a cognitive effect). To do this, we presented participants with the same all audio-visual stream-bounce stimuli as in Experiment 2 (i.e., with varying temporal offsets between the sound and visual coincidence of the targets and the same biased statistics) and tasked them with making a temporal order judgement instead of a perceptual (stream or bounce) judgement. We reasoned that if expectation leads to enhanced multisensory integration (hence increased bounce responses), then the temporal order judgement should be more difficult for high bounce compared with low bounce expectation targets and this would manifest as an increased JND for the former compared to the latter. We in fact found the reverse, and the JND was significantly lower for high (compared with low) bounce expectation targets. Further, we found an unexpected significant difference in the PSE such that the sound was perceived to lead visual coincidence more for high (compared with low) bounce expectation targets.

Our findings in Experiments 1 and 2 demonstrate that an association of target shade with high bounce likelihood sets up an expectation in observers that influences the perceptual resolution of ambiguity. The effect size is small, but we note three things about the association. First, it was established implicitly and, anecdotally, participants remained mostly unaware of the biased stimulus statistics underlying the expectation manipulation. Second, it was established rapidly with only brief exposure to the biased stimulus statistics (less than 10 min). Finally, the association is entirely arbitrary and not driven by an underlying spatiotemporal effect, semantic association, or crossmodal correspondence. This supports previous research suggesting that expectations create predictions about forthcoming sensory events, providing a key mechanism to cope with sensory ambiguity (Costantini et al., 2018).

The finding that the expectation effect was only evident in the high bounce likelihood condition in each experiment (audio-visual vs. visual-only trials in Experiment 1 and small offsets vs. large offsets in Experiment 2) firstly suggests that the effect is not simply one of associating certain stimuli with certain perceptual decisions. If a more general association was occurring, then we would expect the high bounce expectation targets to appear more bounce-like in all conditions in both experiments. Specifically, the high (compared to low) bounce expectation would elicit increased bounce responses in both the visual-only and audio-visual conditions in Experiment 1 and in both the large offset and small offset conditions in Experiment 2. We did not observe this pattern.

More importantly, this finding suggests that the expectation appears to be of a perceptual outcome (a bounce) rather than a stimulus (a sound). This is a distinction that previous research has not typically made. For example, Gekas et al. (2015) and Van Wanrooij et al. (2010) examined expectation in visual search and audio-visual orienting paradigms respectively, and Costantini et al. (2018) found a visuo-tactile cross congruency effect even when a tactile distractor was expected but omitted. While each of these studies show an effect of expectation on perceptual processes, the expectation itself is purely stimulus driven.

This finding that a perceptual rather than stimulus expectation modulates perceptual disambiguation in a stream-bounce display is consistent with our previous findings. Zeljko and Grove (2017b), presented participants with typical stream-bounce stimuli except that the tone, when present, was weak and embedded in auditory noise. Imagined and perceived tones were associated with equivalent bouncing biases, while missed and perceived no-tones were associated with identical streaming biases, suggesting an importance of perception over the actual stimulus. Later, Zeljko and Grove (2021) examined the influence of previous trials on stream-bounce responses and found a strong serial dependence of the previous response but no effect of the previous stimulus and concluded that current perceptual interpretations depend on previous perceptual decisions and not previous stimuli.

Our finding that high bounce expectation leads to improved temporal order discrimination suggests that the increase in bounce responses observed for high (compared to low) bounce expectation targets is not due to expectation-led increases in the likelihood of multisensory integration. This was unexpected given that other studies have found that expectations involving multisensory integration tend to result in improved integration. For example, Gau and Noppeney (2016) found that creating expectations of audio-visual integration by presenting congruent audio-visual phonemes led to increased integration of McGurk stimuli. In a visuo-haptic task, Helbig and Ernst (2007) found that prior knowledge that crossmodal signals belong to a single object promoted integration even if the signals were spatially offset. In an audio-visual study, Fiorini et al. (2021) found electrophysiological evidence of anticipatory multisensorial integration in unimodal brain areas and suggested that this reflected a boost to early stimulus processing and enhanced multisensory integration.

The second finding in Experiment 3, that there is a relative shift in PSE (towards sound leading) for high versus low bounce expectation targets was exploratory, but it is possible that this is related to a well-established asymmetry observed in stream-bounce displays where sounds are offset from visual coincidence. In addition to the finding that small offsets result in a greater proportion of bounce responses than large offsets, offsets with the sound preceding visual coincidence tend to elicit more bounce responses than offsets with sound following coincidence (Sekuler et al., 1997; Watanabe & Shimojo, 2005).

The combined findings that high bounce expectation leads to both increased bouncing and impaired multisensory integration seem contradictory. There is some uncertainty regarding the origin of auditory induced bouncing in terms of bottom-up versus top-down processing and sensory versus cognitive processes (for a review, see Grove et al., 2016). While there is no definitive explanation, there is compelling evidence that cognitive inference plays a substantial role (Zeljko & Grove, 2021), although Maniglia et al. (2012) found evidence supporting a multisensory integration account involving posterior parietal cortex. We suggest two possible explanations. First, it may be that expectation influences cognitive inference in generating bounce percepts rather than enhancing multisensory integration. Further, bounce expectation may lead to some impairment in multisensory integration, but it is small compared to the expectation effect on cognitive inference. Second, it could be that the effects of expectation are simply task related leading to either enhancement or impairment of integration.

In describing the resolution of ambiguity in perception, Brascamp et al. (2008) suggested that competing percepts are determined by an elaborate history of prior perception and Maloney et al. (2005) concluded that sensory systems analyse recent perceptual history to predict the immediate sensory future and these predictions can alter perceptions. Our findings support these ideas and show that past perceptual experience creates expectations that influence multisensory perception, these expectations can be implicitly created, they appear to be expectations about perceptual outcomes rather than sensory stimuli. Finally, in the case of resolving perceptual ambiguity, the expectations effect is an effect on cognitive rather than sensory processes.
